# Interactions between the apolipoprotein E4 gene and modifiable risk factors for cognitive impairment: a nationally representative panel study

**DOI:** 10.1186/s12877-022-03652-w

**Published:** 2022-12-06

**Authors:** Ajay Kolli, Yunshu Zhou, Grace Chung, Erin B. Ware, Kenneth M. Langa, Joshua R. Ehrlich

**Affiliations:** 1 Department of Ophthalmology and Visual Sciences, University of Michigan Medical School, Kellogg Eye Center, 1000 Wall Street, Ann Arbor, MI 48105 USA; 2 Department of Ophthalmology, University of California San Francisco, San Francisco, CA USA; 3 Department of Health Policy and Management, University of Michigan, Ann Arbor, MI USA; 4 Institute for Social Research, University of Michigan, Ann Arbor, MI USA; 5 Division of Medicine, Department of Internal Medicine, University of Michigan Medical School, Ann Arbor, MI USA; 6 Ann Arbor Veterans Affairs Healthcare System, Ann Arbor, MI USA

**Keywords:** Dementia, Cognitive Impairment, Apolipoprotein E4, Gene Environment Interaction

## Abstract

**Background:**

Few studies using rigorous clinical diagnosis have considered whether associations with cognitive decline are potentiated by interactions between genetic and modifiable risk factors. Given the increasing burden of cognitive impairment (CI) and dementia, we assessed whether Apolipoprotein E ε4 (APOE4) genotype status modifies the association between incident CI and key modifiable risk factors .

**Methods:**

Older adults (70+) in the US were included. APOE4 status was genotyped. Risk factors for CI were self-reported. Cognitive status (normal, CI, or dementia) was assigned by clinical consensus panel. In eight separate Cox proportional hazard models, we assessed for interactions between APOE4 status and other CI risk factors.

**Result:**

The analytical sample included 181 participants (mean age 77.7 years; 45.9% male). APOE4 was independently associated with a greater hazard of CI in each model (Hazard Ratios [HR] between 1.81–2.66, *p* < 0.05) except the model evaluating educational attainment (HR 1.65, *p* = 0.40). The joint effects of APOE4 and high school education or less (HR 2.25, 95% CI: 1.40–3.60, *p* < 0.001), hypertension (HR 2.46, 95% CI: 1.28–4.73, *p* = 0.007), elevated depressive symptoms (HR 5.09, 95% CI: 2.59–10.02, *p* < 0.001), hearing loss (HR 3.44, 95% CI: 1.87–6.33, *p* < 0.0001), vision impairment (HR 5.14, 95% CI: 2.31–11.43, *p* < 0.001), smoking (HR 2.35, 95% CI: 1.24–4.47, p = 0.009), or obesity (HR 3.80, 95% CI: 2.11–6.85, *p* < 0.001) were associated with the hazard of incident CIND (compared to no genetic or modifiable risk factor) in separate models. The joint effect of Apolipoprotein ε4 and type 2 diabetes was not associated with CIND (HR 1.58, 95% CI: 0.67–2.48, *p* = 0.44).

**Discussion:**

The combination of APOE4 and selected modifiable risk factors conveys a stronger association with incident CI than either type of risk factor alone.

## Introduction

Dementia affects an estimated 57.4 million individuals worldwide and this number is projected to nearly triple by 2050 [1]. Cognitive impairment typically precedes a diagnosis of dementia, and those with cognitive impairment have an increased risk of dementia compared to cognitively normal adults [2–4]. Because interventions to prevent dementia may be most effective when implemented early in the process of cognitive decline, research is needed to characterize early risk factors for cognitive decline [5].

The Apolipoprotein E ε4 (APOE4) genotype is the strongest genetic risk factor for cognitive decline and late onset dementia; yet, when considered in isolation, it accounts for a relatively small portion of population dementia risk [5, 6]. Prior studies have identified several modifiable risk factors for cognitive decline, including less education, hypertension, type 2 diabetes mellitus (T2DM), depression, hearing loss, vision impairment, smoking, and obesity [5, 7]. However, few studies have considered whether interactions between APOE4 and modifiable risk factors may influence the risk of clinically diagnosed cognitive impairment [8–12].

Prior research on dementia risk reported interactions between APOE4 and smoking in the UK Biobank [8] and between APOE4 and depression in Canadian Study of Health and Aging, [9] while other studies have reported no interaction between smoking, [8] healthy lifestyle, [10, 11] or self-reported hearing loss [12] and polygenic risk for dementia [8, 10, 12, 13] or cognitive decline [11, 12]. In light of data suggesting that APOE4 may be more strongly associated with cognitive impairment than dementia, [14] we hypothesized significant modification by APOE4 status on the association of key modifiable risk factors with incident cognitive impairment.

## Methods

### Study sample

The Aging, Demographics, and Memory Study (ADAMS) was a nationally representative study of a sub-sample of adults age ≥ 70 years recruited from the United States Health and Retirement Study (HRS) from 2000–2002 [15]. The HRS collects a wide array of sociodemographic, health, and economic data that is made publicly available (hrsonline.isr.umich.edu) [16]. Participants returned for study visits approximately every 2 years or until they were diagnosed with dementia. Participants who were cognitively normal at baseline and had ≥ 2 waves of data were included in this analysis. Institutional Review Boards at the University of Michigan and Duke University approved all HRS/ADAMS study procedures. Informed consent was obtained from all study participants or their surrogates. The current secondary analysis of publicly available data was deemed not regulated.

### Cognitive assessment

In ADAMS, a nurse and neuropsychology technician administered cognitive tests and obtained a history on health issues, activities, symptoms, medical history, cognitive or functional impairment, and other contributory factors. Further details of ADAMS methodology has been previously described [15]. Using this information, a provisional diagnosis of cognitively normal, cognitive impairment not dementia (CIND), or dementia was determined by judgement of a consensus panel consisting of a geropsychiatrist, neurologist, neuropsychiatrist, and cognitive neuroscientist. After reviewing medical records, the geropsychiatrist revised the provisional diagnosis as needed. The full consensus panel then arrived at a final diagnosis without knowledge of prior HRS or ADAMS assessments or statuses.

The diagnosis of CIND [15, 17] was defined as: self and/or informant report of problems with cognition or daily activities, or performance on neuropsychological measures that was both below expectation relative to education, reading level, and occupational attainment, and at least 1.5 standard deviations below published norms on at least one test. These are similar to the criteria for a diagnosis of mild cognitive impairment (MCI), but MCI requires both measured declines in cognitive testing and reported problems with cognition or daily activities [18].

### Apolipoprotein E4 (APOE4) genotyping

In ADAMS, APOE4 genotype was assessed via genotyping of a buccal swab DNA sample. The Oragene-250 protocol and saliva kits were used to collect the specimen. Staff at the Center for Inherited Disease Research genotyped the DNA using the Illumina HumanOmni2.5 array (8v1 and 4v1). Quality control was conducted by the Genetics Coordinating Center (University of Washington in Seattle, WA). Carrier status was dichotomized (0 vs 1 or 2 copies) since few participants carried 2 copies of the APOE4 gene.

### Dementia risk factors

Participant characteristics were assessed by self-report (educational attainment [high school or less vs more than high school], hypertension, T2DM, elevated depressive symptoms [Center for Epidemiological Studies Depression score ≥ 4], [15] smoking [ever smoker vs never smoker], age, and gender) or objective measurement (hearing loss [unable to hear rubbing fingers or whispering in either ear], vision impairment [visual acuity < 20/40 in the better seeing eye], obesity [BMI ≥ 35]).

### Statistical analysis

First, a Cox proportional hazard model was constructed with time to incident CIND as the dependent variable and death as a competing risk [19]. Independent variables included: APOE4 status, educational attainment, hypertension, T2DM, elevated depressive symptoms, hearing loss, vision impairment, smoking, obesity, age, and gender. In eight separate iterations of this model, an interaction term between APOE4 and a selected modifiable risk factor was used to test for modification by APOE status. All analyses were conducted using SAS version 9.4 (SAS Institute, Cary, NC, USA). Statistical tests were two-tailed and a significance threshold of *α* = 0.05 was used. The Holm’s Step-Down Procedure was used to adjust for multiple comparisons. Respondents with no event or death were censored at the last wave of the survey in which they participated. Respondents who transitioned from cognitively normal to dementia (without an intervening diagnosis of CIND) were assumed to have developed CIND at the midpoint between being cognitively normal and being diagnosed with dementia [20]. Death was modeled as a competing risk [19].

## Results

Of the 196 participants eligible participants, 181 who had complete data for each of the variables in the models were included in this analysis. Mean (standard deviation [SD]) age was 77.7 [5.1] years and 45.9% were male. Descriptive statistics for the analytic sample are presented in Table 1. Median (interquartile range) follow up duration was 5.0 (2.0-6.6) years.

**Table 1:** Baseline Characteristics of 181 Older Adults by Apolipoprotein E4 Genotype

Characteristic	Overall Sample (*n* = 181)	APOE4 status		
		APOE4: no copies (*n* = 136)	APOE4: ≥ 1 copy (*n* = 45)	*p*-value^a^
Age in Years, mean (SD)	77.7 (5.1)	77.9 (5.3)	77.3 (4.6)	.6701
Male Sex, n (%)	83 (45.9%)	57 (41.9%)	26 (57.8%)	.0641
Vision Impairment, n (%)	24 (13.3%)	18 (13.2%)	6 (13.3%)	.9866
Hearing loss, n (%)	25 (13.8%)	19 (14.0%)	6 (13.3%)	.9145
Elevated depressive Symptoms, n (%)	30 (16.6%)	23 (16.9%)	7 (15.6%)	.8320
More than Highschool Education	37 (20.4%)	23 (16.9%)	14 (31.1%)	.0406
Obesity (BMI ≥ 35 kg/m^2^), n (%)	42 (23.2%)	34 (25.0%)	8 (17.8%)	.3198
Smoking, n (%)	99 (54.7%)	73 (53.7%)	26 (57.8%)	.6319
Hypertension, n (%)	105 (58.0%)	82 (60.3%)	23 (51.1%)	.2793
Type 2 Diabetes Mellitus, n (%)	35 (19.3%)	26 (19.1%)	9 (20.0%)	.8966

*APOE4* Apolipoprotein E4, *BMI* Body Mass Index. Smoking was defined as current or past tobacco smoking

^a^*p*-values are from Wilcoxon Rank-Sum Tests for continuous variables and Chi-Square Tests for categorical variables

**Fig. 1:**
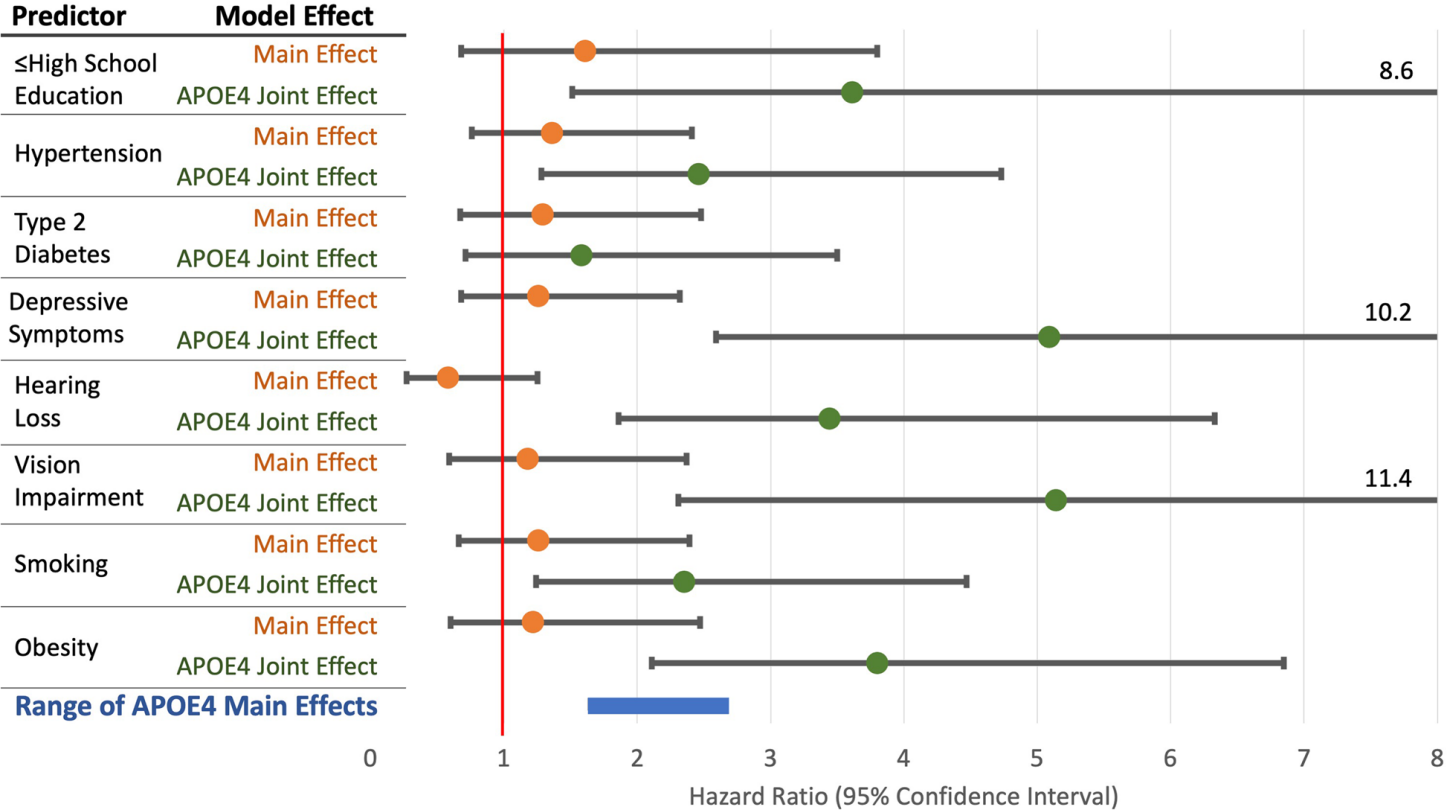
Modifiable Risk Factors and their Joint Effects with Apolipoprotein E4 Genotype as Predictors of Incident Cognitive Impairment No Dementia (CIND)

The APOE4 genotype was independently associated with a greater hazard of CIND in each model (Hazard Ratios [HR] between 1.81–2.66, *p* < 0.05 in all models, Table 2) except the model evaluating educational attainment (HR 1.65, *p* = 0.40). In those with no copies of APOE4, the hazard of CIND was not associated with any of the eight selected modifiable risk factors **(**Fig. 1, Table 2**)**.

**Table 2:** Modifiable Risk Factors, Apolipoprotein E4 Genotype, and their Joint Effects as Predictors of Incident Cognitive Impairment No Dementia (CIND)

**Risk Factor**		**Hazard Ratio**	**95% CI Lower Bound**	**95% CI Upper Bound**	***p*-value**
≤ High School Education	Joint Effect	3.61	1.51	8.61	0.0039
	Risk Factor Effect	1.61	0.68	3.80	0.2811
	APOE4 Effect	1.65	0.52	5.23	0.3986
Hypertension	Joint Effect	2.46	1.28	4.73	0.0068
	Risk Factor Effect	1.36	0.76	2.41	0.2981
	APOE4 Effect	2.65	1.35	5.20	0.0046
Type 2 Diabetes	Joint Effect	1.58	0.71	3.50	0.2611
	Risk Factor Effect	1.29	0.67	2.48	0.4400
	APOE4 Effect	2.51	1.54	4.09	0.0002
Depressive Symptoms	Joint Effect	5.09	2.59	10.02	< .0001
	Risk Factor Effect	1.26	0.68	2.32	0.4622
	APOE4 Effect	1.81	1.09	3.03	0.0229
Hearing Loss	Joint Effect	3.44	1.87	6.33	< .0001
	Risk Factor Effect	0.58	0.27	1.25	0.1659
	APOE4 Effect	1.83	1.13	2.96	0.0141
Vision Impairment	Joint Effect	5.14	2.31	11.43	< .0001
	Risk Factor Effect	1.18	0.59	2.37	0.6369
	APOE4 Effect	1.84	1.12	3.04	0.0168
Smoking	Joint Effect	2.35	1.24	4.47	0.0090
	Risk Factor Effect	1.26	0.66	2.39	0.4797
	APOE4 Effect	2.66	1.24	5.72	0.0120
Obesity	Joint Effect	3.80	2.11	6.85	< .0001
	Risk Factor Effect	1.22	0.60	2.47	0.5840
	APOE4 Effect	1.90	1.15	3.14	0.0117

APOE4: Apolipoprotein E4. BMI: Body Mass Index. Depressive symptoms were defined as Center for Epidemiological Studies Depression score ≥ 4. Smoking was defined as current or past tobacco smoking. Hazard ratios are from 8 separate Cox proportional hazard models with a main effect and a joint effect with APOE4 for the given predictor. Models were fitted with time to incident CIND as the dependent variable and death as a competing risk. Each model was additionally adjusted for age, sex, and each of the other 7 predictors. The bottom row shows the range for the hazard ratio for the main effect of APOE4 across the 7 models (confidence intervals are shown in Table 2).

Conversely, there was a statistically significant interaction between APOE4 and seven of the eight modifiable risk factors in predicting time to CIND **(**Fig. 1**)**. The joint effects of APOE4 and high school or less educational attainment (HR 3.61, 95% CI: 1.51–8.61, *p* = 0.003), hypertension (HR = 2.46, 95% CI: 1.28–4.73, *p* = 0.007), elevated depressive symptoms (HR = 5.09, 95% CI: 2.59–10.02, *p* < 0.001), hearing loss (HR = 3.44, 95% CI: 1.87–6.33, *p* < 0.001), vision impairment (HR = 5.14, 95% CI: 2.31–11.43, p < 0.001), smoking (HR = 2.35, 95% CI: 1.24–4.47, *p* = 0.009), and obesity (HR = 3.80, 95% CI: 2.11–6.85, *p* < 0.001) were associated with incident CIND in separate models. The joint effect of APOE4 and T2DM was not associated with CIND (HR = 1.58, 95% CI: 0.67–2.48, *p* = 0.44).

## Discussion

In this longitudinal study of 181 older adults, APOE4 genotype significantly modified the association between incident CIND and educational attainment, hypertension, elevated depressive symptoms, hearing loss, vision impairment, smoking, and obesity. Given the increasing number of older adults with dementia, detection of early risk factors for cognitive decline and identifying sub-groups for whom early risk factors are most relevant are important research priorities [1–3, 5]. Our results suggest that those with both genetic (e.g., APOE4) and modifiable risk factors may be at particularly high risk for cognitive decline. It is possible that such individuals could benefit from closer monitoring or earlier interventions to slow the progression of cognitive decline (e.g., more intensive cardiovascular risk factor control, exercise, cognitive training, etc.) [5].

Some prior studies have reported interactions between modifiable (e.g., smoking, [8] depression, [9] sleep disordered breathing [23]) and genetic risk for dementia or cognitive decline, while other studies have reported no interaction [10–12]. For example, the interaction between smoking and APOE4 status has been associated with incident dementia in the UK Biobank [8]. On the other hand, no significant interaction between polygenic risk score (excluding the APOE4 allele) and smoking was found in the UK Biobank cohort [8]. As such, it is possible that APOE4 genotype is a stronger modifier of the impact of modifiable risk factors on cognitive health as compared to other gene loci. Further research is needed to determine if there are significant interactions between modifiable dementia or cognitive impairment risk factors and other individual gene loci.

In the present study, there was so significant joint effect between T2DM and APOE4 status. Prior cohort study data have provided mixed epidemiologic evidence for an association between diabetes and cognitive impairment, with some reporting no association [3, 24] while others suggest a significant association [25–27]. As such, it is possible that the lack of a significant joint effect between diabetes and APOE4 status in the present study may be attributable to a lack of association between diabetes and CIND among this study population. Studies with longer follow-up, larger sample size, and assessment of duration of time lived with diabetes, will be important for elucidating the association of T2DM with CIND. Moreover, once one reaches CIND, risk factor interactions could become less important because the risk of progression from CIND to dementia may be high regardless of the presence of other risk factors [2, 3, 20].

The present study assesses risk factors from throughout the life course. For example, educational attainment is more readily modifiable in young adulthood and tends to remain unchanged as participants progress through mid-life and older adulthood, when cognitive impairment and dementia is more prevalent. *The Lancet Commission’s* 2020 Report on Dementia Prevention, Intervention, and Care recommends using a life course framework for understanding risk factors for cognitive decline and dementia [5]. In turn, studies assessing dementia risk factors have typically included risk factors from throughout participants’ life course [8–12]. While some risk factors may no longer be modifiable once individuals reach older-adulthood, early life interventions (e.g. education programs, smoking cessation, diabetes control, etc.) may result in later-life benefits for cognitive health, though considerable interventional research is needed to test this [5]. Further research is also needed to determine whether APOE4 genotype may modulate the effectiveness of early interventions to prevent cognitive decline [28].

The present study has several strengths compared to prior studies, including longitudinal follow up (median 5.0 years), consensus panel diagnoses of cognitive status, genotyping of all subjects, robust covariate adjustment, inclusion of 8 key CIND risk factors, and use of CIND as an outcome, which may have a stronger association with APOE4 than dementia [14]. Prior studies reporting no interaction differed from the present study based on lack of robust cognitive status assessment and/or assessment of polygenic risk rather than APOE4 status.

## Limitations

Studies with a larger sample may be better suited for assessing interactions, subgroup analyses (e.g., by ancestry), and dose-response effects by number of APOE4 allele copies. Moreover, some null findings may have been due to large standard errors related to our relatively small sample size. Nevertheless, with a sample size of 181 participants, the present study detected significant interactions between APOE4 genotype and seven of the eight modifiable risk factors that we investigated, suggesting the sample was adequate to test the study’s hypothesis. Additionally, some participant characteristics were ascertained by self-report (educational attainment, hypertension, T2DM, elevated depressive symptoms, and smoking). Use of objective measures (e.g. blood pressure or hemoglobin A1c) could provide additional useful information about the relationship between chronic disease, APOE4 genotype, and cognitive impairment.

### Conclusion

The combination of APOE4 genotype and modifiable risk factors conveys a stronger association with incident CIND than either type of risk factor alone. If these findings are confirmed, they may be useful for risk stratification, prognostication, and targeting of early interventions.

## Data Availability

The HRS collects a wide array of sociodemographic, health, and economic data that is made publicly available (hrsonline.isr.umich.edu).

## Abbreviations

APOE4: Apolipoprotein E ε4
T2DM: Type 2 diabetes mellitus
ADAMS: Aging Demographic and Memory Study
HRS: Health and Retirement Study
CIND: Cognitive impairment, no dementia
BMI: Body mass index. HR: Hazard Ratio
